# Pushing the boundaries. Concurrent Hodgkin lymphoma and breast cancer treatment with preservation of pregnancy: A case report

**DOI:** 10.1016/j.gore.2022.100937

**Published:** 2022-01-31

**Authors:** Charlotte LeJeune, Daan Dierickx, Hans Wildiers, Lore Lannoo, Kristel Van Calsteren, Vincent Vandecaveye, Björn Menten, Joris Vermeesch, Frédéric Amant

**Affiliations:** a Gynecological Oncology, Department of Oncology, KU Leuven, Leuven, Belgium; b Department of Hematology, University Hospitals Leuven, Leuven, Belgium; c Department of General Medical Oncology, University Hospitals Leuven, Leuven Belgium; d Department of Development and Regeneration, Division Woman and Child, Clinical Department of Obstetrics and Gynecology, University Hospitals Leuven, Leuven, Belgium; e Department of Radiology, University Hospitals Leuven, Leuven, Belgium; f Department of Biomolecular Medicine, Ghent University Hospitals, Ghent, Belgium; g Department of Human Genetics, University Hospitals Leuven, Leuven, Belgium; h Department of Gynecologic Oncology, University Hospitals, Leuven, Belgium; i Surgical Oncology, the Netherlands Cancer Institute, Amsterdam, the Netherlands

**Keywords:** Pregnancy, Breast cancer, Hodgkin lymphoma, Case report

## Abstract

**Background:**

Breast cancer and hematological cancers are the most commonly diagnosed malignancies during pregnancy. This case report is the first to describe the ultimate challenge to preserve a pregnancy while the expectant mother is diagnosed and treated simultaneously for two concurrent primary malignancies, a stage IIA Hodgkin lymphoma and pT2N0(Sn) breast cancer.

**Clinical case:**

A 36-year-old pregnant primigravida underwent a routine non-invasive prenatal test at 14 weeks and 4 days of gestation. Genome-wide sequencing was used and revealed an aberrant DNA/chromosome copy number profile among which a strong 2p-gain, possibly related to a maternal malignancy. Physical examination showed an enlarged cervical lymph node and ultrasound guided biopsy confirmed the diagnosis of a nodular sclerosing classical Hodgkin lymphoma subsequently staged as an early stage, unfavorable (IIA) Hodgkin lymphoma. Whole body magnetic resonance imaging for further staging also indicated a suspicious nodule in the right breast. Further investigation resulted in the concurrent diagnosis of a pT2N0(Sn) invasive ductal adenocarcinoma. Patient underwent a mastectomy with sentinel lymph node biopsy at 15 weeks and 5 days of gestation, followed by 4-weekly chemotherapy administration, consisting of doxorubicin, bleomycin, vinblastine and dacarbazine (ABVD). Pregnancy went further relatively uncomplicated and fetal assessment was reassuring during pregnancy. Due to fever of unknown origin and preterm labor, a cesarean section was performed on a gestational age of 35 weeks and 4 days. Oncological treatment was completed after delivery with involved-field radiation therapy for the Hodgkin lymphoma. Completion of systemic treatment for breast cancer consisted of docetaxel/cyclophosphamide chemotherapy, and anti-hormonal treatment in the form of ovarian function suppression and letrozole.

**Conclusion:**

Here we show for the first time that two concurrent primary malignancies can be treated successfully during pregnancy with respect to maternal and fetal chances. Motivated modifications of breast cancer treatment (mastectomy instead of lumpectomy, AVBD instead of epirubicin-cyclophosphamide chemotherapy), allowed treatment of both cancers during pregnancy. Final treatment was administered after delivery.

## Highlights

• This is the first reported case of two concurrent primary cancers during pregnancy.
• The multidisciplinary tumor board was critical in defining the best treatment.
• Modifications to the standard-of-care should always be motivated.
• Both cancers were treated during pregnancy with surgery and chemotherapy.
• The result was a good maternal and obstetric outcome.

## 1 Introduction

About 1 in every 1000–2000 pregnancies is complicated by cancer and this incidence has increased in the last decades (de Haan et al., 2018). The large-scale implementation of non-invasive prenatal testing (NIPT) to screen for fetal aneuploidies results in an increase of oncological diagnoses during pregnancy, revealed by aberrant NIPT results (Amant et al., 2015). Breast cancer (BC) and hematological cancers are the most commonly diagnosed malignant diseases during pregnancy (de Haan et al., 2018). BC is the most common cancer diagnosis, accounting for 39% of malignancies diagnosed during pregnancy (de Haan et al., 2018) with an incidence of 2.4–7.3 per 100,000 pregnancies (Loibl et al., 2015). Hodgkin lymphoma (HL) is the most common hematological malignancy diagnosed during pregnancy with an estimated incidence of 2–6 per 100,000 pregnancies (Maggen et al., 2019).

To the best of our knowledge, this is the first reported case of two concurrent primary malignancies diagnosed during a single pregnancy, describing a good obstetric and oncological outcome. The case report was approved by the local ethics committee for publication. The patient provided written informed consent to be included in this case report.

## 2 Case presentation

**Fig. 1:**
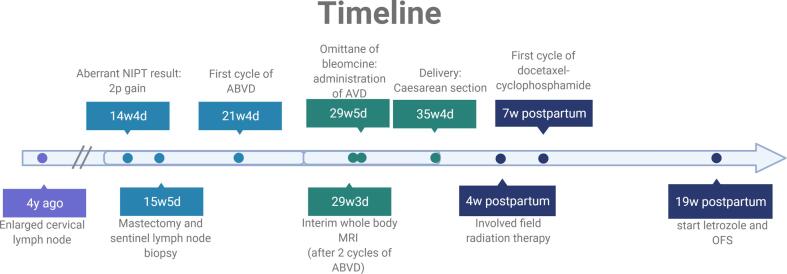
Timeline of interventions during the pregnancy and postpartum.

At 14 weeks and 4 days of pregnancy, a 36-year-old woman (gravida 4, para 3) underwent a routine NIPT screening. Apart from 3 cesarean sections, her medical history was unremarkable. During her previous pregnancy, she noticed an enlarged cervical lymph node at 36 weeks pregnant (Fig. 1). Ultrasound guided lymph node biopsy was inconclusive at that time due to insufficient sample. A NIPT was performed with a normal result. Since the clinical aspect of the lymph node became non-suspect on ultrasound after the delivery, no further examinations were performed.

**Fig. 2:**
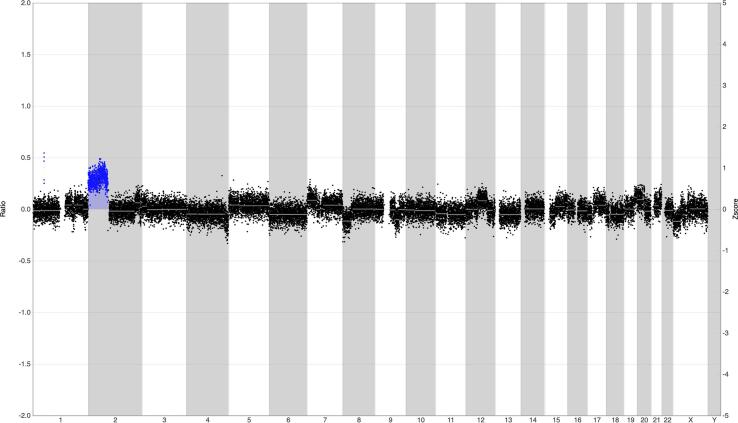
The chromosome copy number profile after NIPT analysis for chromosomes 1–22, X and Y shows several (mosaic) aberrations on several chromosomes, among which a clear chromosome 2p gain. Such a profile is highly indicative for the presence of a (maternal) malignancy.

**Fig. 3:**
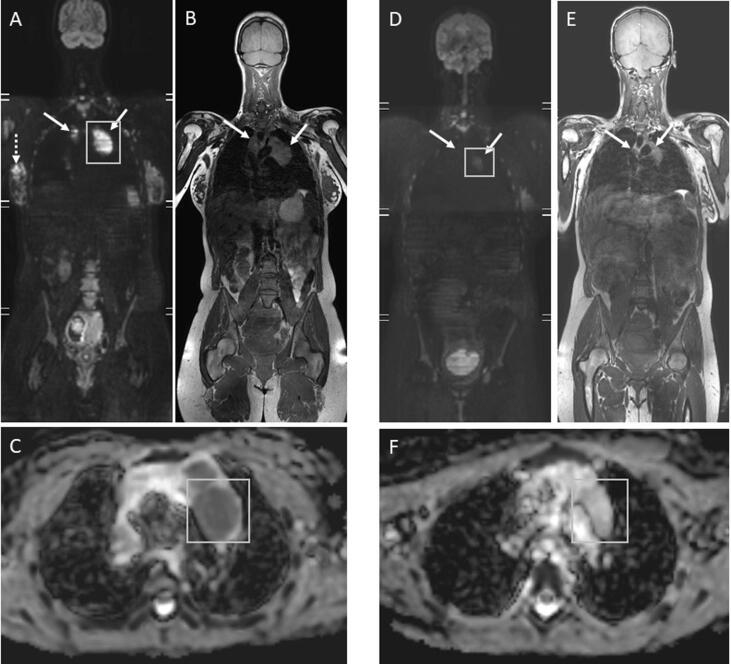
Whole body diffusion-weighted magnetic resonance (WB-DWI/MRI) in pregnant patient with aberrant noninvasive prenatal test (NIPT): (A) b1000 DWI shows enlarged and bright mediastinal lymphadenopathies (arrows) with anatomical correlate at (B) T1-weighted image indicative of lymphoma and a bright nodular lesion (dashed arrows) in de the right breast suspicious of breast cancer. (C) Corresponding apparent diffusion coefficient (ADC) map of the mediastinal lymphadenopathy (green box) shows value of 0.00089 mm^2^/sec confirming malignancy. Interim WB-DWI/MRI after 2 cycles of chemotherapy and right mastectomy shows (D) signal substantial loss at b1000 DWI and partial volume regression at (E) T1-weighted image of the mediastinal lymphadenopathies (arrows) corresponding to (F) a substantially increased ADC of 0.00191 mm^2^/sec indicating substantial necrosis induction and thus excellent response of the lymphoma.

During the present pregnancy, genome-wide NIPT showed an aberrant DNA copy number profile. Several chromosomes showed imbalances, among which a clear 2p-gain (Fig. 2), possibly related to a maternal malignancy. Given the continued presence of the enlarged cervical lymph node, the ultrasound was repeated showing a heterogeneous nodular cervical mass in region IV, measuring 42 × 26 mm. Whole body diffusion weighted magnetic resonance imaging (WB-DWI/MRI) was performed which showed multiple, bilateral periclavicular and mediastinal adenopathies, without evidence for organ- or bone marrow involvement (Fig. 3). A biopsy of the cervical lymph node showed a nodular sclerosing classical Hodgkin lymphoma (CD30^+^ CD15^int^ CD20^-^) (NSCHL). The patient was diagnosed with early stage, unfavorable (IIA) NSCHL.

Staging WB-DWI/MRI also indicated a nodule in the right breast, suspect of lymphoma localization, but additional investigations were planned due to this atypical location. Follow-up breast ultrasound showed a poorly delineated hypoechogenic mass, measuring 27 × 15 mm, in the axillary tail, BIRADS 5, with absence of axillary adenopathies. Core needle biopsy showed a grade 1 to grade 2, hormone receptor positive, Her2 negative, invasive ductal adenocarcinoma (IDA) not otherwise specified stage pT2N0(Sn). In summary, at 15 weeks pregnancy, this asymptomatic pregnant patient was diagnosed with NSCHL and hormone sensitive BC. Since NIPT results were not interpretable for fetal anomalies, amniocentesis was proposed but parents opted for ultrasonographic follow-up only.

Patient underwent a right-sided mastectomy with sentinel node procedure with injection of Technetium in a one-day protocol at 15 weeks and 5 days of pregnancy. Use of patent blue dye was avoided due to the concurrent pregnancy. Frozen section pathology showed 2 negative lymph nodes. Additional pathological examination after surgery confirmed the diagnosis of a pT2N0(Sn) grade 2 luminal B like IDA.

At 21 weeks and 4 days of gestation, the patient received the first of 4 cycles of 4-weekly chemotherapy ABVD (doxorubicin 25 mg/m^2^, bleomycin 10 mg/m^2^, vinblastine 6 mg/m^2^, dacarbazine 375 mg/m^2^). WB-DWI/MRI after two cycles showed a major metabolic remission (Fig. 3). Bleomycin was subsequently omitted from the chemotherapeutic regimen given the favorable interim response and to decrease the risk of interstitial pulmonary fibrosis. The chemotherapy was well-tolerated. Ultrasonographic fetal follow-up occurred every two weeks and showed a normal growth on the 70th percentile without structural abnormalities.

At 35 weeks and 4 days of gestation, the patient was admitted to the hospital with a fever of unknown origin and painful contractions. AVD (doxorubicin 25 mg/m^2^, vinblastine 6 mg/m^2^, dacarbazine 375 mg/m^2^) cycle 4 day 15 was postponed. Due to clinical deterioration and history of previous cesarean sections, she underwent a repeat cesarean section, and gave birth to a healthy son weighing 2875 g with an APGAR score of 9 and 10 at subsequently 1 and 5 min. On day 1 postpartum, patient developed an acute respiratory distress syndrome, attributed to an pneumocystis jiroveci pneumonia, and was admitted to the intensive care unit for 3 days followed by a full recovery.

One month postpartum, patient received involved-field radiation therapy of all affected lymph nodes, with a total dose of 30 Gray (Gy) in 15 fractions of 2 Gy; radiotherapy of the breast was not required because of mastectomy and absence of risk factors. Due to young age and T2 status, adjuvant taxane chemotherapy for the BC was considered. An additional molecular prognostic panel (MammaPrint®) showed a genomic high risk score, and patient subsequently started chemotherapy 1 week after completion of the consolidation radiotherapy, receiving an additional 4 cycles of docetaxel (75 mg/m^2^) – cyclophosphamide (600 mg/m^2^) in a 3-weekly regimen. After completion of the chemotherapy, adjuvant anti-hormonal treatment in the form of ovarian function suppression and letrozole was started.

Positron emission tomography and computed tomography (PET-CT) after termination of the adjuvant chemotherapy showed complete metabolic remission. Follow-up after 6 months was reassuring, with no evidence for relapse of either the BC or HL. At 6 months, the boy had a normal growth and weight gain (percentile 50) and normal development. He was treated with propranolol for an infantile hemangioma on the forehead, with good response.

## 3 Discussion and conclusions

This case report describes the ultimate challenge to preserve a pregnancy while the expectant mother is diagnosed and treated simultaneously for a stage IIA lymphoma and pT2N0(Sn) BC. The main challenge is to preserve maternal outcome by defining which treatment modality is most needed and how both HL and BC treatment can be combined during pregnancy. In this patient, we opted for a mastectomy rather than a lumpectomy in order to facilitate HL radiotherapy postpartum and because of an increased risk of secondary breast cancer in HL survivors. The combination of ABVD was considered the best systemic treatment due to its efficacy in both concurrent malignancies. Cancer treatment was completed after delivery with involved-field radiation therapy for HL. Completion of systemic treatment for BC consisted of docetaxel/cyclophosphamide chemotherapy, and anti-hormonal treatment in the form of ovarian function suppression and letrozole.

The NIPT is used for the detection of fetal aneuploidies and analyze cell-free DNA (cfDNA) circulating in maternal plasma. In cancer patients, part of the cell-free DNA originates from the tumor and a majority of cancers are characterized by chromosomal copy number alterations, which can be detected by genome-wide sequencing NIPT (Lenaerts et al., 2021, Lannoo et al., 2021). Aberrant NIPT-results may reveal an occult maternal malignancy in asymptomatic patients (Amant et al., 2015).

The option to terminate the pregnancy was offered to the couple based on the gestational age and the experimental nature to treat two cancers during pregnancy. However, they preferred to preserve the pregnancy based on the available evidence of oncological treatment during pregnancy. Chemotherapy during the first 12 weeks of pregnancy is associated with an increased risk for congenital malformations in the fetus, whereas the incidence of congenital malformations in patients who received chemotherapy in the second or third trimester of pregnancy is comparable to the incidence of congenital malformations in the general population, approximately 3% (van Gerwen et al., 2021). Children prenatally exposed to chemotherapy have normal cognitive and cardiac outcomes and a general development within normal ranges, provided that the chemotherapy was administered after 12 weeks of pregnancy (Vandenbroucke et al., 2020, Amant et al., 2015, Vandenbroucke et al., 2017). Over the last decades, increased awareness of the feasibility and safety of chemotherapy during pregnancy resulted in more patients receiving antenatal treatment (de Haan et al., 2018). Oncological treatment during pregnancy should deviate as little as possible from the standard treatment of non-pregnant patients and follow guidelines as closely as possible to preserve maternal prognosis.

Local treatment of early stage HL has evolved to combined-modality treatment including involved-field radiation therapy (Maggen et al., 2019, Eichenauer et al., 2018). Local treatment of early BC involves surgery with or without adjuvant radiotherapy, with the majority (60–80%) of patients receiving breast sparing surgery (Cardoso et al., 2019). In our patient, breast sparing surgery was technically feasible based on tumor size and location. However, after multidisciplinary consultation, mastectomy was advised because breast radiotherapy would technically complicate lymphoma radiotherapy due to partial overlapping of radiation fields and because of an increased risk of secondary breast cancer in HL survivors after thoracic radiotherapy. The cumulative incidence of secondary breast cancer in HL survivors by the age of 50 is 35% with the most important risk factor being younger age at treatment (Bakkach et al., November 2020).

Current standard treatment of early stage classical Hodgkin Lymphoma (CHL) consists of a combined-modality treatment including a limited number of chemotherapy cycles, usually 2–4 cycles of ABVD. Survival outcomes in pregnant patients with HL treated with ABVD do not differ significantly from non-pregnant controls (Maggen et al., 2019). Systemic treatment of BC involves chemotherapy and targeted-therapy and anti-hormonal therapy if required. The most frequently used chemotherapy regimens in BC treatment contain anthracyclines and/or taxanes. Sequential anthracycline/taxane-based regimens significantly reduce BC mortality and are considered to be standard of care for the majority of patients. When the standard of care is administered, prognosis of breast cancer during pregnancy is similar to non-pregnant patients with breast cancer (Loibl et al., 2015). Epirubicin, a 4′-epimer of doxorubicin, is the most frequently used anthracycline due to the more favorable hematologic and cardiologic toxicity profile, but doxorubicin is therapeutically equivalent (Khasraw et al., 2012). The dosage of doxorubicin (25 mg/m^2^) in treatment for HL results in a cumulative dose of 200 mg/m^2^ after 4 cycles of ABVD. This is comparable to the cumulative dose of 240 mg/m^2^ of doxorubicin administered at 60 mg/m^2^ for 4 cycles in combination with cyclophosphamide (600 mg/m^2^; AC) in adjuvant BC treatment. However, dose intensity of doxorubicin in ABVD is lower than in AC. In high-risk BC patients, dose dense treatment, consisting of the same chemotherapy dose over a shorter interval, leads to better survival in comparison to conventionally dosed chemotherapy regimens (Loibl et al., 2015). Both chemotherapeutic regimens can be administered during pregnancy (de Haan et al., 2018, Maggen et al., 2019), so the choice of chemotherapeutic regimen was determined by the oncological problem. Due to its lower anthracycline dose density, ABVD could be considered as a suboptimal chemotherapeutic regimen in the adjuvant treatment of BC. However, in this case, ABVD was considered the best option due to its relative efficacy in treating both concurrent malignancies. It was decided upfront that additional taxane and cyclophosphamide could still be added postoperatively, knowing that the latter by itself is already an effective adjuvant regimen.

Treatment of CHL has evolved to a response-adapted treatment, where interim use of functional imaging after 2 cycles of chemotherapy gives an early indication of chemosensitivity. PET-CT is the clinical standard in lymphoma. A positive interim PET can direct early treatment intensification to improve prognosis (Eichenauer et al., 2018, Ansell, 2020). A negative interim PET can lead to de-escalation strategies to minimize treatment-related toxicity with no negative impact on outcome (Ansell, 2020, Gunther et al., 2020). In our case, interim PET-CT was replaced by WB-DWI/MRI. In pregnant patients, WB-DWI/MRI is considered a non-irradiating alternative to PET-CT for diagnosing and staging of cancers and lymphoma (Han et al., 2018). Specifically for lymphoma, WB DW-MRI has shown a concordance of up to 99.4% for detecting nodal and extranodal lymphoma and allows a reliable interim response evaluation (Vandecaveye et al., 2021, Mayerhoefer et al., 2014). In our case, a favorable interim response on WB-DWI/MRI after two cycles of ABVD resulted in the removal of bleomycin from the chemotherapy schedule, reducing the risk of maternal pulmonary toxicity with added benefit of decreasing fetal chemotherapy exposure.

Here, with this unique case, we show that two concurrent primary malignancies can be treated successfully during pregnancy with respect to maternal and fetal outcome. Decision making via a multidisciplinary tumor board was critical to define the best local and systematic treatment options based on standard treatment paradigms and the consequences for each of the two malignancies as well as safety in pregnancy. Indeed, with motivated modifications of breast cancer treatment (mastectomy instead of lumpectomy, AVBD instead of EC chemotherapy), both cancers could be treated during pregnancy and final treatment was administered after delivery.

## Ethics approval and consent to participate

Written informed consent was obtained from the patient for publication of this case report and accompanying images. A copy of the written consent is available for review by the Editor-in-Chief of this journal on request.

## Funding

The work was supported by Kom op tegen kanker (Stand up to Cancer), the Flemish cancer society [grant number KotK_KUL/2019/11815/1]

## CRediT authorship contribution statement

**Charlotte LeJeune:** Writing – original draft. **Daan Dierickx:** Investigation, Writing – review & editing. **Hans Wildiers:** Investigation, Writing – review & editing. **Lore Lannoo:** Investigation, Writing – review & editing. **Kristel Van Calsteren:** Investigation, Writing – review & editing. **Vincent Vandecaveye:** Investigation, Writing – review & editing. **Björn Menten:** Investigation, Writing – review & editing. **Joris Vermeesch:** Investigation, Writing – review & editing. **Frédéric Amant:** Conceptualization, Supervision, Writing – review & editing.

## Declaration of Competing Interest

The authors declare that they have no known competing financial interests or personal relationships that could have appeared to influence the work reported in this paper.
